# USING TRANSDISCIPLINARY CO-PRODUCTION TO GAIN INSIGHTS INTO SPATIAL JUSTICE AND AGE-FRIENDLY CITIES

**DOI:** 10.1093/geroni/igad104.1043

**Published:** 2023-12-21

**Authors:** Patty Doran

**Affiliations:** The University of Manchester, Manchester, England, United Kingdom

## Abstract

Globally, a popular policy approach to address the two interrelated challenges of population ageing and urbanisation has been to adopt the World Health Organization’s Age-Friendly City framework, a model that can help to identify and address barriers to the well-being and participation of older people. This paper reports early findings from the ‘Ageing in Place in Cities’ project. The research was conducted from a critical gerontology perspective and embedded a spatial justice perspective by centring the concepts of equity, diversity and co-production in our underpinning theoretical framework. Discussing findings from seven case study cities (all who were early adopters of the Age-Friendly Cities framework), this paper discusses how a transdisciplinary co-production approach has been used to gain greater understanding of age-friendly initiatives in each city. Using mixed methods, we first developed descriptive profiles for each city using both census and other local demographic data to explore how the populations have changed over time, and narratives and policy documents detailing the initiatives used to support ageing in place. Second, we conducted semi-structured interviews with a variety of actors in each city to examine how the cities delivered age-friendly change. We integrated the data using a comparative case study approach to draw out insights across the cities. Our findings reveal the role of key leaders and stakeholders in age-friendly cities and will be of interest to policy makers and academics interested in population ageing and urban change. Further, our transdisciplinary co-production methods present a novel approach to conducting age-friendly research.

